# Rumen Bacterial Community Responses to Three DHA Supplements: A Comparative In Vitro Study

**DOI:** 10.3390/ani15020196

**Published:** 2025-01-13

**Authors:** Jianmin Zou, Genna Ba, Dian Wang, Mengmeng Li, Shaohong Jin, Chong Chen, Wei Tan, Jian He, Hengsheng Du, Pengjie Wang, Yinhua Zhu

**Affiliations:** 1 School of Food and Biological Engineering, Hefei University of Technology, Hefei 230601, China; 2021030064@mail.hfut.edu.cn; 2 Department of Nutrition and Health, China Agricultural University, No. 17 Tsinghua East Road, Haidian District, Beijing 100083, China; zb20223311103@cau.edu.cn (S.J.); chenchong409@cau.edu.cn (C.C.); 3 Engineering Research Center of Bio-Process, Ministry of Education, Hefei University of Technology, Hefei 230601, China; 4 Inner Mongolia Yili Industrial Group, Co., Ltd., Hohhot 010110, China; bgn@yili.com (G.B.); tanwei01@yili.com (W.T.); 5 Inner Mongolia Youran Dairy Group Limited, Hohhot 010010, China; wd@yourandairy.com; 6 State Key Laboratory of Animal Nutrition and Feeding, College of Animal Science and Technology, China Agricultural University, Beijing 100193, China; limeng2021@cau.edu.cn; 7 National Center of Technology Innovation for Dairy, Hohhot 010110, China; hejian@yili.com (J.H.); dhs@yourandairy.com (H.D.)

**Keywords:** in vitro digestion, DHA supplements, rumen bacterial community, DHA bioaccessibility

## Abstract

The aim of this study was to investigate the loss of docosahexaenoic acid (DHA) from three supplements (two powders and one oil) after digestion (rumen and gastrointestinal) and their effects on the number and composition of rumen bacteria, using an in vitro approach. The concentration of supplements has a significant impact on the DHA loss rate and algal oil exhibited the highest rate of loss, but bioaccessibility was not significantly different from the other supplements. 16S rRNA sequencing showed that three DHA supplements altered the bacterial composition of in vitro batch cultures inoculated with rumen microorganisms from cows, and caused changes in the relative abundance of important bacterial phyla, families, and genera. DHA supplements altered the abundance of bacterial species, including *Prevotella*, *Ruminobacter*, *Succiniclassicum*, *Succinivibrio*, *Lachnospiraceae*, and *Muribaculaceae*. Importantly, these changes may be associated with the ruminal response in biohydrogenation. Algal oil has the most significant impact on rumen microbiota by reducing the richness and diversity of rumen microbiota, and significantly altering the composition of multiple important microbiota.

## Simple Summary

Docosahexaenoic acid (DHA) supplements are added to cow feed for the production of DHA milk, and many derivative products have been gradually developed to meet commercial needs. The loss of these commercial supplements in the rumen and their effects on rumen microorganisms are rarely reported. This study aimed to investigate the loss of DHA from three supplements (two powders and one oil) after total digestion and their effects on the number and composition of rumen bacteria, using an in vitro approach. The results showed that algae oil had the highest rate of loss, but bioaccessibility was not significantly different from the other supplements; three DHA supplements altered the bacterial composition of in vitro batch cultures inoculated with rumen microorganisms from cows, and caused changes in the relative abundance of main bacterial phyla, families, and genera. Algal oil has the most significant impact on rumen microbiota by reducing the richness and diversity of rumen microbiota, and significantly altering the composition of multiple important microbiota. We also observed that some rumen bacteria may be involved in the ruminal response in biohydrogenation to the addition of marine lipids, but further research is necessary to confirm their actual role in ruminal lipid metabolism.

## 1. Introduction

Docosahexaenoic acid (DHA) is an essential polyunsaturated fatty acid (PUFA) with high nutritional value, which can influence brain and retina development, prevent cardiovascular disease, and participate in many important physiological processes [1,2]. DHA is naturally present at low concentrations in milk, and the addition of marine lipids to the diet of ruminants has proven useful to increase the level of DHA in milk [3,4].

Previous studies have reported that adding different sources of marine lipids, such as fish oil [5], microalgal powder [4], and microalgal oil [3], as DHA supplements to the diet can result in the production of milk with high levels of DHA. However, fish oil is no longer adapted to the needs of intensive dairy farming because of its inherently uncontrollable source, high price, and risk of contaminants [6], and because its large-scale use may result in consumer sustainability issues, such as environmental pollution and damage to marine ecosystems [7].

*Schizochytrium* sp. are microalgae rich in ω3-PUFAs such as DHA. Compared with fish oil and other microalgae, *Schizochytrium* sp. have potential as DHA supplements, and many derivative products have been gradually developed to meet commercial needs, such as high-purity algal oil, rumen-passing algal powder, etc. [3]. *Schizochytrium* sp. has been shown to be effective in increasing the DHA content of milk [8,9]. The amount of DHA supplementation added to the diet generally does not exceed 5% of the total feed [2,4,8,9,10]. The daily intake of DHA by cows may reach several tens of grams; however, the DHA translocated to milk remains limited. It has been reported that most DHA is lost during rumen digestion, with loss rates reaching 60–90% [2,11,12]. DHA loss during rumen digestion is a major factor influencing DHA conversion, and various additives may affect DHA loss in the rumen [3]. Thus, investigating the rumen loss associated with different supplements is essential. The process by which unsaturated fatty acids in the diet are converted to saturated fatty acids by rumen bacteria is known as biohydrogenation [13]. Most studies suggest that biohydrogenation is a major factor in the loss of polyunsaturated fatty acids (PUFAs) from the rumen [2,14], with rumen microorganisms playing a central role. Recent studies suggest that PUFA-rich supplements alter the core rumen bacterial community [15], thereby affecting the biohydrogenation of UFAs. In addition, PUFAs may affect both the viability and metabolism of methanogenic microbes [16], and inhibit the growth of certain cellulolytic and butyrate-producing bacteria [17]. Changes in bacterial communities induced by PUFAs may disrupt the normal physiology of the rumen, negatively impacting fatty acid metabolism, cellulose digestion [17], crude protein degradation, volatile fatty acid production, and methane emission [18]. Alterations in rumen microorganisms are crucial to ruminant health and development. However, the effects of commercial supplements, particularly those containing DHA, on rumen microorganisms have been rarely reported. Therefore, it is crucial to explore the effects of these supplements on rumen flora for their selection and application. The loss of these commercial supplements, especially those containing DHA, in the rumen and their effects on rumen microorganisms are rarely reported.

Therefore, the aim of this study was to use in vitro methods to investigate DHA loss after digestion (rumen and gastrointestinal) of three *Schizochytrium*-based supplements (two algal powders and one algal oil), as well as their effects on the abundance and composition of rumen bacteria. At the same time, the changes in microbial abundance were used to identify the microbial communities that may participate in DHA biohydrogenation after adding supplements, providing some reference for subsequent research and the development of DHA rumen-passing products.

## 2. Materials and Methods

### 2.1. Animals and Materials

We selected five healthy Holstein cows with permanent rumen fistulas to serve as donors for rumen inoculation. All fistulated cows were housed together with regular lactating cows in the same barn and were fed three times a day. The composition and nutritional levels of the basal diet are detailed in Table 1. The three types of DHA supplements [standard algal powder (SAP), processed algal powder (PAP), and algal oil (AO)] were supplied by Xiamen Huisheng Biotechnology Co., Ltd. [8]. The DHA content of the supplements is shown in Table 1. The three supplements used were regular algal powder, rumen-bypass algal powder, and high-concentration DHA algal oil.

### 2.2. In Vitro Rumen Fermentation

This trial was conducted in vitro using a 2 × 4 factorial design: two concentrations (0.5 or 1 mg DHA/mL rumen fluid) and four treatments (SAP, PAP, AO, and a control without DHA additive, with the supplement pre-mixed with dry matter), with at least three replicates per treatment. The bioinformatic analysis of rumen microbiota was performed using a single DHA concentration (1 mg/mL). Batch cultures of rumen microorganisms were conducted as described previously [19,20,21]. Microbial culture was performed in Hungate tubes, using rumen inocula collected from five cannulated cows. All animals were fed the same high-concentrate total mixed ration, which was offered at estimated maintenance energy requirements [22]. Rumen fluid was obtained before feeding and strained through four layers of sterile gauze. The rumen fluids were mixed (1:2) with Menke buffer (Table S1) [20]. Hungate tubes, pretreated with substrate dry matter (150 mg) and different concentrations of DHA supplements [19], were incubated under anaerobic conditions at 39 °C. Gas accumulation was prevented through the regular timed insertion of a hypodermic needle in the rubber stopper. The reaction was stopped after 24 h, and samples were collected for the determination of ruminal loss rates and DNA extraction.

### 2.3. Determination of Digestive Loss Rate and Bioaccessibility of DHA

In vitro simulation of gastrointestinal digestion was performed according to the method of Vinyard and Wang [1,23]. We added 2 mL 0.9 M HCl (mixed pepsin 20 mg) to the tube after in vitro fermentation, resealed the tube, and incubated at 39 °C for 1 h. Different concentrations of DHA supplements [19] were incubated under anaerobic conditions at 39 °C. Gas accumulation was prevented through the regular timed insertion of a hypodermic needle in the rubber stopper. The reaction was stopped after 24 h, and samples were collected for the determination of ruminal loss rates and DNA extraction. Following incubation, the mixture was neutralized with 2 mL 0.9 M NaOH. Following neutralization, we added 3 mL of concentrated intestinal buffer solution (0.5 M KH_2_PO_4_, 16.5 g/L bile salt from cattle, 52.8 g/L pancreatin from cattle, and 9.64 g/L CaCl_2_). The tubes were mixed, resealed, and incubated at 39 °C for 24 h. After removal of the fermentation tube, digestion was immediately terminated by inactivating the enzymes, and all digested chyme was collected.

The digested chyme was divided into two parts: bioaccessibility and digestive loss rate. After centrifugation of the digesta at 4000 g for 5 min, followed by another round of centrifugation at 21,000 g for 20 min, the upper fat layer and lower sediment were discarded, and the middle liquid was taken as the micellar fraction and freeze-dried for determination of the bioaccessibility and digestive loss rate.

### 2.4. Analysis of Rumen DHA Content

Rumen DHA content was analyzed as previously described [1]. Freeze-dried powder was placed in a 15 mL tube, and 2 mL toluene was added, followed by 4 mL acetyl chloride/methanol (1:9) solution. The test tube was filled with nitrogen gas, shaken well, and incubated at 80 °C for 2 h in a water bath. This was then transferred to a 50 mL centrifuge tube and rinsed three times with 3 mL sodium carbonate. After centrifugation at 4500 g for 5 min, we collected the supernatant and analyzed the DHA content using gas chromatography–flame ionization detection and an SP-2560 column (100 m, 0.25 mm ID, 0.25 μm film; Sigma–Aldrich, St. Luis, MO, USA). The temperature of the sampler was 270 °C and the temperature of the detector was 260 °C. The temperature program was set as follows: the initial temperature was 130 °C for 5 min; this increased at a rate of 4 °C/min until it reached 240 °C; then, it was maintained for 20 min. The carrier gas was nitrogen, with a split ratio of 100:1 and an injection volume of 1.0 μL. The percentage of DHA lost during digestion relative to the total amount of DHA was defined as the digestive loss rate of DHA. The DHA content in the digesta was measured, and the digestive loss rate of DHA in the sample was calculated using the following equation:

Ruminal loss rate (%) = (C _initial_ − C _ruminal fermentation_)/C _initial_ × 100 (1)

where C _ruminal fermentation_ is the DHA content in the rumen fermentation (mg) and C _initial_ is the DHA content in the sample before rumen fermentation (mg).

Digestion loss rate (%) = (C _initial_ − C _fermentation_)/C _initial_ × 100 (2)

where C _fermentation_ is the DHA content in the rumen–gastrointestinal digesta (mg) and C _initial_ is the DHA content in the sample before rumen fermentation (mg).

Bioaccessibility (%) = C _micellar fraction_/C _initial_ × 100 (3)

where C _micellar fraction_ is the DHA content in the digested micellar fraction (mg) and C _initial_ is the DHA content in the sample before rumen fermentation (mg).

### 2.5. DNA Extraction and Polymerase Chain Reaction Amplification

Freeze-dried samples were thoroughly mixed before DNA extraction. Total microbial genomic DNA was extracted from samples using the PF Mag-Bind Stool DNA Kit (Omega Bio-tek, Nocross, Georgia, USA). The quality and concentration of DNA were determined by 1.0% agarose gel electrophoresis and a NanoDrop^®^ ND-2000 spectrophotometer (Thermo Scientific Inc., Waltham, MA, USA) and kept at −80 °C prior to use. The hypervariable region V3–V4 of the bacterial 16S rRNA gene was amplified with primer pairs 338F (5′-ACTCCTACGGGAGGCAGCAG-3′) and 806R (5′-GGACTACHVGGGTWTCTAAT-3′) [24] by an ABI GeneAmp^®^ 9700 PCR thermocycler. The polymerase chain reaction (PCR) mixture included 4 μL 5× Fast Pfu Buffer, 2 μL 2.5 mM dNTPs, 0.8 μL Forward Primer (5 μM), 0.8 μL Reverse Primer (5 μM), 0.4 μL Fast Pfu polymerase, 0.2 μL BSA, 10 ng template DNA, and deionized distilled water to a final volume of 20 µL. PCR amplification cycling conditions were as follows: initial denaturation at 95 °C for 3 min, followed by 27 cycles of denaturation at 95 °C for 30 s, annealing at 55 °C for 30 s, extension at 72 °C for 45 s, and single extension at 72 °C for 10 min and then at 10 °C until halted by the user. All samples were amplified in triplicate. The PCR product was extracted from 2% agarose gel and purified. Then, it was quantified using the Quantus™ Fluorometer (Promega, Madison, WI, USA).

### 2.6. Illumina Sequencing

Purified amplicons were pooled in equimolar amounts and paired-end sequenced on an Illumina PE300 platform (Illumina, San Diego, CA, USA) according to the standard protocols of Majorbio Bio-Pharm Technology Co., Ltd. (Shanghai, China) The raw sequencing reads were deposited into the NCBI Sequence Read Archive (SRA) database.

### 2.7. Data Processing

Raw FASTQ files were de-multiplexed using an in-house perl script, quality-filtered by fastp version 0.19.6 [25], and merged by FLASH version 1.2.1 [26]. The optimized sequences were clustered into operational taxonomic units (OTUs) using UPARSE 11 [27] with 97% sequence similarity. The most abundant sequence for each OTU was selected as a representative sequence. The taxonomy of each OTU representative sequence was analyzed by RDP Classifier version 2.13 [28] against the 16S rRNA gene database (e.g., Silva v138) using a confidence threshold of 0.7.

### 2.8. Statistical Analysis

Data were expressed as mean ± standard deviation, with at least 3 replicates per treatment. Statistical analysis and plotting of experimental results were performed using SPSS 27.0 and GraphPad Prism software (9.5.1). Multiple comparisons of means were conducted using one-way ANOVA followed by Duncan’s multiple range test. Differences between means were considered significant at *p <* 0.05. Bioinformatic analysis of the rumen microbiota was carried out using the Majorbio Cloud platform (https://cloud.majorbio.com, accessed on 3 April 2024.), with 5 replicates per treatment. Based on the OTUs information, rarefaction curves and alpha diversity indices, including observed OTUs, Chao1 richness, the Shannon index, and Good’s coverage, were calculated with Mothur version 1.30.2 [29]. The similarity among the microbial communities in different samples was determined by principal coordinate analysis (PCoA) based on Bray–Curtis dissimilarity using Vegan version 2.4.3. The permutational multivariate analysis of variance test was used to assess the percentage of variation explained by the treatment, along with its statistical significance, using Vegan version 2.4.3. The linear discriminant analysis (LDA) effect size [28] (http://huttenhower.sph.harvard.edu/LEfSe, accessed on 3 April 2024.) was performed to identify the significantly abundant taxa (phyla to genera) of bacteria among the different groups (LDA score > 2, *p* < 0.05). At different taxonomy levels, the relative abundance of communities was plotted by GraphPad Prism version 9.5.1.

## 3. Results

### 3.1. Disappearance of DHA from Cultures of Rumen Digestion

**Figure 1:**
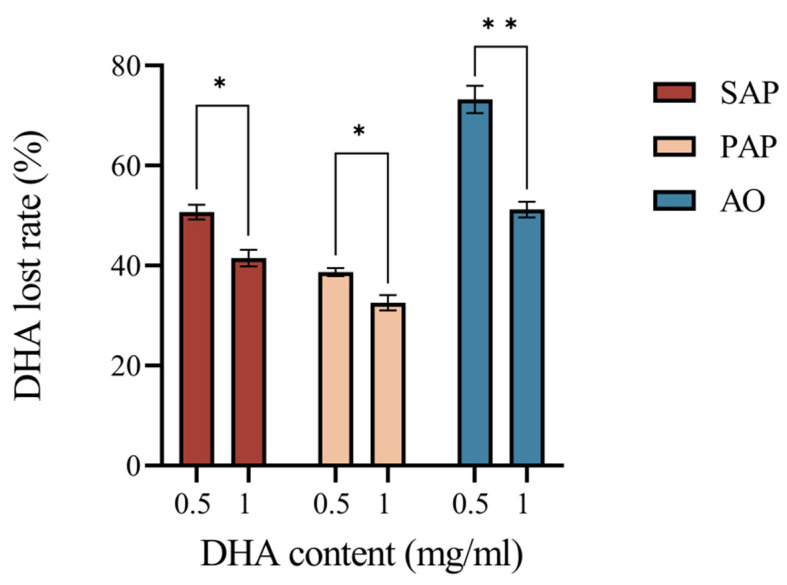
DHA loss rate of different groups after 24 h in vitro ruminal fermentation. Significant difference levels: * *p* < 0.05; ** *p* < 0.01. Error bars show the standard deviation.

The ruminal loss rates of the three supplements after 24 h of simulated rumen fermentation in vitro are shown in Figure 1. The three supplements showed different DHA disappearance after in vitro rumen fermentation, with AO showing the highest rate of loss (from 73.25% to 51.21%). Concentration affects the rate of DHA loss, with AO being more sensitive to concentration changes, which began to decrease when the concentration was increased from 0.5 to 1 mg/mL, and the same trend was observed for PAP (from 38.67% to 32.59%) and SAP (from 50.70% to 41.51%) (*p <* 0.05).

After DHA was digested in the rumen, it moved on to the next stage of gastrointestinal digestion. We determined the bioaccessibility and gastrointestinal digestion loss rate of the three supplements. Bioaccessibility refers to the proportion of DHA that is released from the food matrix into the intestinal lumen during digestion and formed into mixed micelles before subsequent absorption into the small intestine [1]. It represents the amount of DHA that is absorbed. The digestive loss rate is the amount of DHA lost after rumen–gastrointestinal digestion. In Table 2, the digestion loss rate of AO (76.80%) was significantly higher than that of SAP (68.43%) after simulating the total digestion process in vitro (*p* < 0.05). The digestion loss rate of PAP (65.51%) was not significantly different from either AO or SAP (*p* > 0.05). Nevertheless, no significant difference was observed in the bioaccessibility of the three supplements (19.24%, 22.11%, and 23.62%, respectively), indicating that AO was more easily absorbed than SAP and PAP.

### 3.2. Bacterial Diversity Analysis

#### 3.2.1. Overview of Sequencing Data

**Figure 2:**
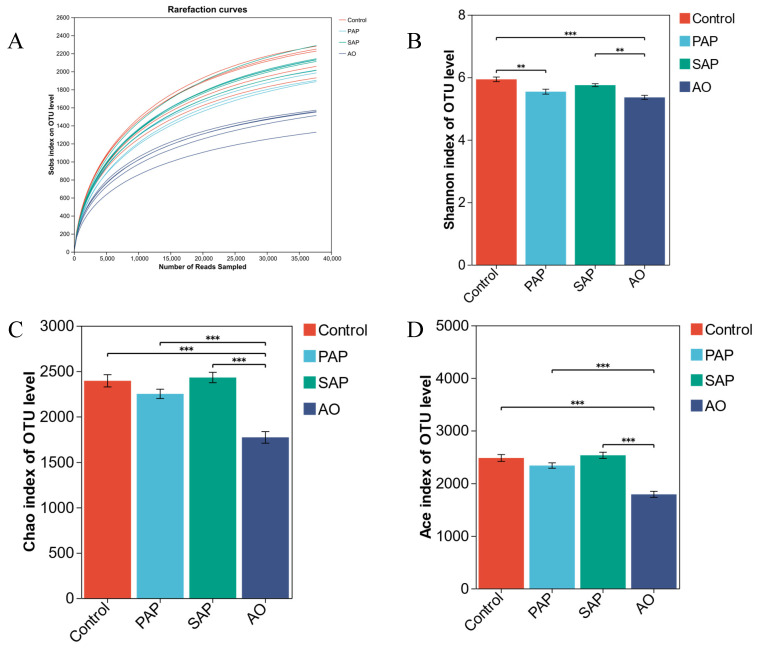
Rarefaction curves and alpha diversity in different treatment groups. (**A**): Rarefaction curve; Kruskal–Wallis H test for the Shannon index (**B**), Chao index (**C**), and Ace index (**D**). Significant difference levels: ** *p* < 0.01; *** *p* < 0.001. Error bars show the standard deviation. (**A**) Rarefaction curves indicate a near-complete sampling of the community. (**B**) The Shannon index suggests that AO and PAP significantly reduced the diversity of rumen microflora. (**C**,**D**) The Chao and Ace indexes indicate that algal oil significantly reduced rumen microbial richness. There was no significant difference (*p* > 0.05) between the two types of algal powder and the control group.

High-throughput sequencing of bacterial 16S rRNA genes generated 1,374,458 raw reads. These sequences were optimized through clustering to retain 37,698 sequences for each sample, which allowed identification of up to 2281 OUTs. A similar number of sequences was found in previous analyses of the rumen bacterial community [19,30]. The rarefaction curves showed clear asymptotes and Good’s coverage for the observed OTUs was 99.12% (Figure 2A), which together indicate a near-complete sampling of the community.

#### 3.2.2. Alpha Diversity of Rumen Microbiota

The Chao, Ace, and Shannon indexes are commonly applied to evaluate the microbial community in terms of its diversity and richness [31]. The Shannon index for AO and PAP was significantly lower than for the controls (*p* < 0.05) (Figure 2B.). This suggests that AO and PAP significantly reduced the diversity of rumen microflora. The Chao (Figure 2C) and Ace (Figure 2D) indices of the AO group were significantly lower than those of the other treatment groups (*p* < 0.05), indicating that algal oil significantly reduced rumen microbial richness. There was no significant difference (*p* > 0.05) between the two types of algal powder and the control group.

#### 3.2.3. Beta Diversity of Rumen Microbiota

**Figure 3:**
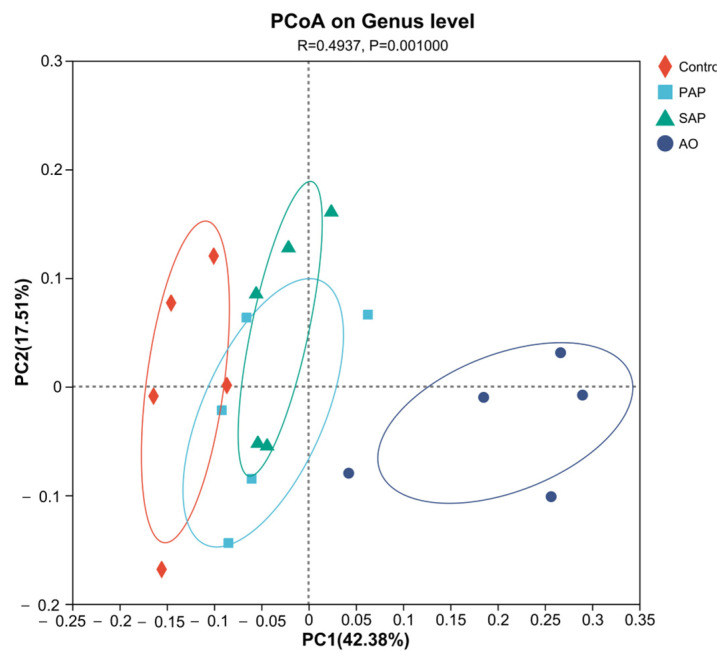
Beta diversity analysis by PCoA, based on the abundance of genera in the microbial community using Bray–Curtis dissimilarity. The different treatment groups were as follows: standard algal powder (SAP), processed algal powder (PAP), and algal oil (AO). Beta diversity analyses indicated significant changes in the bacterial microbiota when adding different supplements.

The Bray–Curtis distance-based PCoA (PCo1 variance = 42.38%, PCo2 variance = 17.51%) visualized the beta diversity of the microbiome, revealing microbial community differences of the three supplements after fermentation in vitro. PCoA showed that more-distant groups had less similarity in species composition [31]. There was a significant separation between the control group and each treatment group (Figure 3). The distance of the AO group was further, and the distance between the SAP and PAP groups was closer, and the SAP group even had overlaps with PAP. These beta diversity analyses indicated significant changes in the bacterial microbiota when adding different supplements.

### 3.3. Composition of Rumen Microbiota

**Figure 4:**
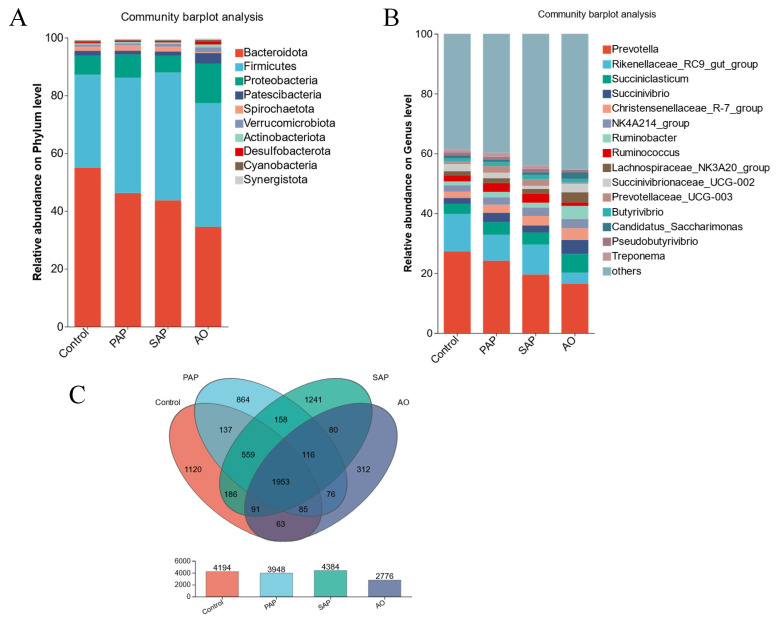
Composition of rumen microbiota. The different treatment groups were as follows: standard algal powder (SAP), processed algal powder (PAP), and algal oil (AO). Distribution of and difference in the bacterial community structure in different treatment groups at the phylum (**A**) and genus (**B**) levels, respectively. The column chart demonstrated that distinct samples exhibited a similar dominant species at the phylum or genus level, yet exhibited disparate relative abundances. (**C**) Venn diagram showing the unique and shared OTUs of the bacterial communities across different groups.

Venn’s research was focused on the analysis and display of the number of unique and common species in different groups, with the objective of understanding the changes in species under different influencing factors. Figure 4C shows that 1953 OTUs were jointly present in rumen fluid across all four groups. In addition, each sample had OTUs specific to other samples, with a range of unique OTUs varying from 312 to 1241. The AO group had the lowest number of OTUs, and the addition of algal oil reduced the number of species in the rumen microbiota.

All treatment groups were subjected to analysis of microbial community composition at the phylum and genus levels. The column chart demonstrated that distinct samples exhibited a similar dominant species at the phylum level, yet exhibited disparate relative abundances (Figure 4A). Eight phyla exhibited relative abundances > 0.5% in all samples. Taxonomic entities with relative abundances > 5% were considered major taxa and subjected to further analysis. In each treatment group, the composition of the bacterial community showed the usual distribution pattern [19,30,32,33]. *Bacteroidetes* (55.04–34.58%), *Firmicutes* (42.74–32.14%), and *Proteobacteria* (13.65–6.56%) were the dominant phyla. In comparison to the control group, all three treatment groups exhibited a notable reduction in the relative abundance of *Bacteroidetes* and an increase in the relative abundance of *Firmicutes* (*p* < 0.05).

At the genus level (Figure 4B), *Prevotella* (27.35–16.52%) and *Rikenellaceae-RC9_gut_group* (12.43–3.66%) were the dominant genera, followed by *Succiniclassicum* (6.25–3.35%) and *unclassified_f_Maurbaculaceae* (6.97–2.22%). These genera belong to three phyla (*Bacteroidetes*, *Firmicutes*, and *Proteobacteria*). AO downregulated the relative abundance of *Prevotella*, *Rikenellaceae_RC9_gut_group*, and *unclassified_f__F082*, while upregulating the relative abundance of *Succiniclasticum, unclassified_f__Muribaculaceae*, *Ruminobacter*, and *Succinivibrio*.

### 3.4. Species Difference Analysis of Rumen Microbiota

**Figure 5:**
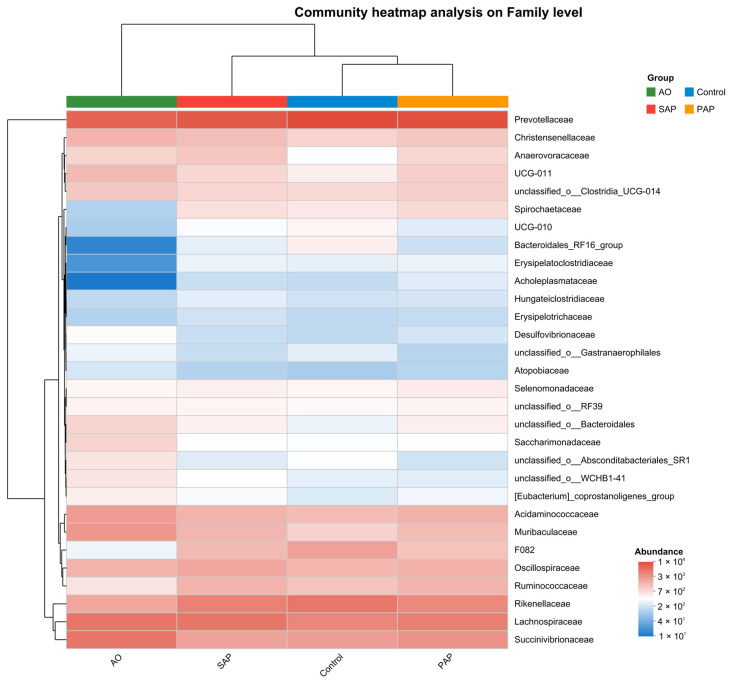
Community heatmap analysis on the family level. The different treatment groups were as follows: standard algal powder (SAP), processed algal powder (PAP), and algal oil (AO). Horizontal coordinates are treatment groups, vertical coordinates are species names, and the color blocks in the heatmap represent the relative abundance of a certain family, with the values represented by the color gradient on the right side of the figure. Heatmap analysis indicated that the SAP, PAP, and control groups had a similar composition of dominant species, whereas the AO and control groups had differences in the composition of dominant species.

**Figure 6:**
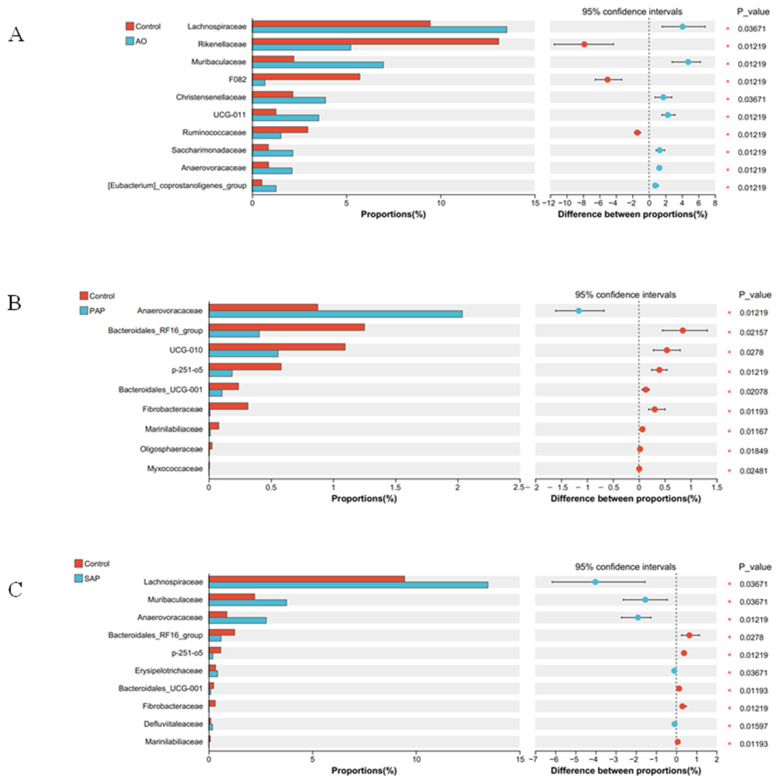
Differences in the bacterial families between each treatment group and the control group. The different treatment groups were as follows: standard algal powder (SAP), processed algal powder (PAP), and algal oil (AO). Statistical analysis was performed by the Wilcoxon rank-sum test. *n* = 5, in each group. * *p* < 0.05. (**A**) Difference between AO and control group; (**B**) Difference between PAP and control group; (**C**) Difference between SAP and control group. Bar charts showing differences in mean relative abundance of the same species between groups, with labels indicating whether the differences are significant or not. The top 10 bacterial families are displayed with a total mean.

To investigate how bacterial composition changed among different groups, a heatmap based on the top 20 species of bacterial profiles was presented (Figure 5). The color blocks in the heatmap represent the relative abundance of a certain family. The SAP, PAP, and control groups had a similar composition of dominant species, whereas the AO and control groups had differences in the composition of dominant species. The predominant families of each group included *Succinivibrionaceae*, *Lachnospiraceae*, *Rikenellaceae*, and *Prevotellaceae*. These changes were confirmed by continuing to analyze species differences at the family level. The Wilcoxon rank-sum test was used to analyze microorganisms with significant differences between the control and treatment groups. It is possible that these differing microorganisms are keystone species that respond to environmental shifts (Figure 6). There were specific responding families between each treatment group and the control group, but there were also some families that were present in different treatment groups.

The *Lachnospiraceae*, *Muribaculaceae*, and *Anaerovoracaceae* families significantly increased their relative abundance in both the AO and SAP groups (Figure 6A,B, *p* < 0.05), but only the *Anaerovoracacae* family responded similarly in the PAP group (Figure 6C, *p* < 0.05).

## 4. Discussion

We first evaluated the disappearance of DHA during in vitro digestion of three DHA supplements. Compared to algal powder, PUFA-rich oil may be accessible to rumen microorganisms [3,4,34]. The availability of nutrients from microalgae to rumen animals can be limited by the presence of robust cell walls or other cell coverings composed of cellulose [35]. Some studies have reported that disruption enhances traits associated with the extent of ruminal fermentation [35]. These changes may be attributed to the destruction of ruminally undegradable cell wall compounds and the increased accessibility of rumen microbiota to fermentable intracellular compounds, which are expected to be released through cell disruption. This may explain the higher loss rate of AO compared to algal powder. Additionally, the effect of DHA concentration on the rate of loss was found to be significant in the present study, which is consistent with previous research [11]. In a previous study [11], a higher degree of biohydrogenation for DHA and EPA was observed when fish oil was incubated at less than 1 mg/mL of ruminal fluid. However, as the fish oil concentration increased in cultures, the degree of biohydrogenation for DHA and EPA decreased [11]. It is hypothesized that two factors contribute to the reduced biohydrogenation. On one hand, high concentrations of DHA have been shown to exacerbate rumen microbial changes [19], which in turn inhibit biohydrogenation. On the other hand, the chemically bound form of DHA from algal sources is primarily in the form of triglycerides, which must be converted into free fatty acids before undergoing biohydrogenation by rumen microbes [2]. However, there is a limit to the hydrolysis of triglycerides in the rumen, which is influenced by multiple factors [36]. An increase in DHA concentration may result in more triglyceride-type DHA entering the gastrointestinal tract, potentially decreasing the rate of DHA loss. In our study, no significant difference in bioaccessibility was observed among the three supplements, despite a 20%-higher rate of AO loss in the rumen compared to SAP and PAP. This result may be attributed to the robust cell walls of SAP and PAP, as unbroken cells may limit the release of certain nutrients [35]. Therefore, when designing or manufacturing DHA supplements, it is important to consider whether DHA can be targeted for release in the gastrointestinal tract to enhance its bioaccessibility.

Supplementation of PUFAs in in vitro rumen digestion affects nutrient degradation, fermentation characteristics, and microbial composition [13,18,19,37]. In this study, three supplements significantly altered the composition of rumen microbiota, and different forms and types of supplements affect rumen microbes at different levels. Supplements influence the α-diversity indices, (ACE, Chao, and Shannon), which reflect microbial diversity and richness. The richness and diversity of rumen microbiota are important indicators of normal biochemical processes in the rumen [16]. Higher diversity and richness are hallmarks of a good rumen microbial ecosystem, which means that physiological processes can be effectively regulated to provide protection to the host [38,39]. Our findings revealed that AO significantly reduced the richness and diversity of rumen microorganisms, which also means that algal oil destroyed the rumen microbial ecosystem and interfered with the normal physiological functions of the rumen. This phenomenon may be attributed to the toxicity of DHA for rumen microorganisms [17]. DHA has bacteriostatic and bactericidal effects [40], and it can inhibit the growth of various microorganisms. The effect of the two types of algal powder on diversity was small, indicating that encapsulation of DHA can reduce the interference of UFAs such as DHA on the rumen microbial ecosystem.

At the phylum level, after adding supplements changes in the bacterial community structure were evident. The addition of three supplements exhibited a notable reduction [35] in the relative abundance of *Bacteroidetes* and an increase in the relative abundance of *Firmicutes*. Supplementation seemed to drive the shift from the *Bacteroidetes* to *Firmicutes*. This may have been due to the DHA in the supplements, and a similar phenomenon has been found in previous studies [19]. The increased relative abundance of *Firmicutes* and decreased abundance of Bacteroidetes, which can result in a higher F/B ratio, is related to higher feed utilization and daily milk fat yield in cows [37,41]. In our study, three supplements increased the relative abundance of the F/B ratio, suggesting that the addition of these supplements to animal diets has the potential to improve energy utilization efficiency and could be used for milk fat content.

*Prevotella* is the most prevalent genus within the Bacteroidetes, and it is the genus with the highest relative abundance in the rumen microbiota [42]. *Prevotella* plays a significant role in ruminant digestion, as it is responsible for the digestion of proteins, starch, and polysaccharides [38,43]. In addition, research indicates that it plays a significant role in reducing greenhouse gas emissions from ruminants [44]. *Prevotella* may also be involved in the biohydrogenation of DHA, with some strains suggested to play a role in ruminal C18 biohydrogenation, specifically in C18:0 formation [45]. Additionally, *Prevotellaceae_UCG-001* was found to be involved in long-chain hydrogenation in the study by Wang et al. [46]. The proportion of *Prevotella* decreased with the three supplements. One possible reason is that the DHA contained in the supplements inhibits the growth of *Prevotella* [19]. Intriguingly, the relative abundance of *Succiniclasticum*, *Ruminobacter*, and *Succinivibrio* significantly increased in the AO group but the same trend was not observed for SAP and PAP. They play an important role in carbohydrate degradation and volatile FA production [18,37,47]: *Succiniclasticum* is able to use succinate to produce propionate [19], *Ruminobacter*, an amylolyic bacterium, can degrade proteins, and is positively correlated with gas production [47], and *Succinivibrio* is involved in utilizing fermentation products to produce succinate, lactate, acetate, and formate and transfer hydrogen away from methanogenesis [18,48]. It is inevitable that alterations in the fundamental microbial community will result in modifications to rumen homeostasis. AO had the most significant effect on the major phyla, which lends support to its effect on diversity.

Intriguingly, supplementation induced changes in the abundance of some bacteria associated with biohydrogenation. In our study, the *Lachnospiraceae* family demonstrated the most substantial response to supplementation. The *Lachnospiraceae* family comprises the main genera with confirmed biohydrogenating ability, including *Butyrivibrio* and *Pseudobutyrivibrio* [2,19,34,49]. The *Lachnospiraceae* family may contain many strains involved in the biohydrogenation of FAs. The addition of PUFAs may affect the abundance of biohydrogenating bacteria. In pure cultures, Jeyanathan et al. suggested that DHA addition had a major effect on the growth of *Butyrivibrio proteoclasticus* P18, which was the only strain identified in the rumen bacteria with the ability to hydrogenate DHA [50]. Similar responses were also observed in the study carried out by Carreno et al., who found significant reductions in the low abundance of *Butyrivibrio* and *Pseudobutyrivibrio* after PUFA addition [19]. Some studies have also suggested that algae as a source of DHA supplementation did not influence the abundance of *Butyrivibrio* spp. [34,49,50]. In our work, there was no significant difference in the abundance of *Butyrivibrio* and *Pseudobutyrivibrio* between the three treatment groups and the control group, which was similar to the results of Zhu et al. [34]. However, the relative abundance of *Lachnospiraceae* in the AO and SAP groups increased. A probable explanation is a higher response and tolerance of other *Lachnospiraceae* to DHA compared with *Butyrivibrio* and *Pseudobutyrivibrio.*

At the family and genus levels, many bacteria responded to the three DHA supplements, such as *Prevotella*, *Ruminobacter*, *Succiniclassicum*, *Succinivibrio*, *Lachnospiraceae*, *Muribaculaceae*, and *Anaerovoracaceae*, which suggests a potential association with ruminal DHA biohydrogenation. Nevertheless, it is difficult to identify with which step of rumen biohydrogenation these genera are directly or indirectly involved. Additional research is needed to provide insight into the metabolic pathways of these microbial groups.

In brief, the distinct supplement forms exerted a notable influence on the destabilization of DHA within the rumen, and this was observed to feed back to the rumen microbes. Of the three supplements, the AO form of DHA, which is more readily hydrogenated, had the most pronounced effect on microorganisms. AO significantly reduced rumen microbial abundance and diversity, and reduced the abundance of important digestive bacteria, such as *Prevotella*, greatly affecting rumen ecological stability. Although there are some advantages of AO in terms of bioavailability [2], the way AO is used still needs to be carefully considered. Additionally, our study focuses on the changes in rumen flora induced in vitro. The next step could involve using in vivo experiments to further investigate these changes, considering that bacterial adaptations, dosage differences, and the more complex digestive environments and longer digestive cycles of in vivo conditions may prevent the changes observed in vitro from being replicated. Furthermore, whether these factors reduce or negate the effects of the supplements should be explored through additional experiments and validation.

## 5. Conclusions

After in vitro digestion, AO showed the highest rate of digestion loss, but there was no significant difference in bioaccessibility, suggesting better utilization. AO, SAP, and PAP altered the bacterial community of in vitro batch cultures inoculated with rumen microorganisms from cows, and caused changes in the relative abundance of major phyla, increased the relative abundance of *Firmicutes*, and reduced the relative abundance of *Bacteroidetes*. At the family and genus levels, the three DHA supplements also had a significant impact, such as on *Prevotella*, *Ruminobacter*, *Succiniclassicum*, *Succinivibrio*, *Lachnospiraceae*, *Muribaculaceae*, and *Anaerovoracaceae*. These bacteria responded to the three DHA supplements, which suggests a potential association with ruminal DHA biohydrogenation. Further research is necessary to examine the metabolic activity of these bacteria and determine if they are truly involved in biohydrogenation in cows. Among the three supplements, AO had the most significant impact on rumen microbiota, and was also the supplement with the highest rumen loss rate. It reduced the richness and diversity of the rumen microbiota, and significantly altered the relative composition of multiple major microbiotas, posing a significant challenge to maintaining the stability of rumen microbiota systems.

## Figures and Tables

**Table 1:** Ingredient and nutrient composition of the dairy cows’ diet, and DHA content in supplements.

Items	Content (%)
Corn	23.32
Soybean meal	13.88
Alfalfa hay	11.96
Cottonseed fuzzy	3.71
Corn silage	29.71
Sodium bicarbonate	1.04
Soybean hulls ground	4.55
Brewer’s grain wet	5.25
Corn gluten meal	0.97
Bergafat	0.47
High cow pre-mix	2.05
Calcium carbonate	0.37
Corn gluten feed dry	1.37
Flavor	0.02
Nutritional level	%, DM
Dry matter	51.64
Metabolizable energy Mcal/kg DM	2.64
Crude protein	17.19
Non-fibrous carbohydrate	29.29
Acid detergent fiber	18.63
Starch	27.01
Total fat	5.07
Calcium	0.87
Phosphorus	0.47
DHA content in supplements	
DHA content	mg/g
AO	692.89
SAP	218.27
PAP	246.08

Standard algae powder = SAP, Processing algae powder = PAP, Algal oil = AO.

**Table 2:** Bioaccessibility and digestive loss rate of DHA in the three types of supplements under in vitro digestion conditions.

Sample	SAP	PAP	AO	*p*-Value
Digestion loss rate	68.43 ± 2.43 ^b^	65.51 ± 7.91 ^ab^	76.80 ± 1.66 ^a^	0.03
Bioaccessibility	19.24 ± 3.07	22.11 ± 3.85	23.62 ± 1.71	0.46

Results are presented as means ± SD of three replicates. Different superscript letters in each line indicate statistically significant differences (lower-case letters represent *p* ≤ 0.05). The initial DHA concentration was 1 mg/mL in all treatment groups.

## Data Availability

The original contributions presented in this study are included in this article/the Supplementary Materials. Further inquiries can be directed to the corresponding authors.

## Supplementary Materials

The following supporting information can be downloaded at: https://www.mdpi.com/article/10.3390/ani15020196/s1, Table S1: Menke buffer formulated solution components.
