# Supplementary material for: Rumen Bacterial Community Responses to Three DHA Supplements: A Comparative In Vitro Study
Source: Animals (Basel). 2025 Jan 13;15(2):196. doi: 10.3390/ani15020196 (PMC11758605; doi:10.3390/ani15020196)
Supplement: Supplementary file 1 [file animals-15-00196-s001.zip › animals-3326922-supplementary.pdf]

Menke buffer preparation method

The addition ratio of rumen fluid to Menke buffer is 1:2, and the following is an example of configuring 1 L of buffer[1]. The composition of the Menke buffer preparation solution is shown in Supplementary Table S1.

Supplementary Table S1. Menke buffer formulated solution components.

Solution A	
$CaCl_2 \cdot 2H_2O$	1.32 $g \cdot L^{-1}$
$MnCl_2 \cdot 4H_2O$	1.00 $g \cdot L^{-1}$
$CoCl_2 \cdot 6H_2O$	0.10 $g \cdot L^{-1}$
$FeCl_3 \cdot 6H_2O$	0.80 $g \cdot L^{-1}$
Solution B	
NH_4HCO_3	4 $g \cdot L^{-1}$
$NaHCO_3$	35$g \cdot L^{-1}$
Solution C	
$Na_2HPO_4 \cdot 12H_2O$	9.45 $g \cdot L^{-1}$
KH_2PO_4	6.2 $g \cdot L^{-1}$
$MgSO_4 \cdot 7H_2O$	0.6$g \cdot L^{-1}$
Solution D	
NaOH	4 mL1 $mol \cdot L^{-1}$
$NaS \cdot 9H_2O$	625 mg
L-Cysteine hydrochloride	625 mg
Distilled water	95 mL

208.1 mL of solution B, 208.1 mL of solution C, 0.1 mL of

solution A and 520.2 mL of distilled water were mixed and the solution was boiled in a microwave oven for about 10 min. The solution was stirred, kept at 39 °C, and CO_2 gas was continuously introduced for about 1 h. The pH of the buffer was measured, if the pH was lower than 6.8, the pH was adjusted to 6.8 by adding NaOH (2 mol/L) and then the reducing agent solution (liquid D) was added to remove any residual oxygen from the solution. Artificial rumen fermentation broth was prepared by mixing rumen fluid with artificial saliva in a ratio of 1:2.

References

[1] Zhou, Y.Q.; Cheng, Y.F.; Zhu, W.Y. The Process and Matters Needing Attention of *in vitro* Fermentation of Rumen Microorganisms. *Bio-101* **2021**, e2003663, doi:10.21769/BioProtoc.2003663.
